# Restoration of surfactant activity by polymyxin B in lipopolysaccharide-potentiated injury of immature rabbit lungs

**DOI:** 10.1038/s41598-020-79679-z

**Published:** 2021-01-08

**Authors:** Andrea Calkovska, Marie Haegerstrand-Björkman, Tore Curstedt

**Affiliations:** 1 Laboratory for Surfactant Research, Department of Molecular Medicine and Surgery, Karolinska Institutet at Karolinska University Hospital, Stockholm, Sweden; 2 Present Address: Department of Physiology, Jessenius Faculty of Medicine, Comenius University, Mala Hora 4C, 036 01 Martin, Slovakia

**Keywords:** Respiratory distress syndrome, Paediatric research

## Abstract

During postnatal adaptation pulmonary surfactant may be inactivated by lipopolysaccharide (LPS). We evaluated the effect of surfactant therapy in combination with antibiotic polymyxin B (PxB) in double-hit model of neonatal lung injury. Surfactant (poractant alfa, Curosurf) was exposed to smooth (S) LPS without/with PxB and tested in captive bubble surfactometer. Preterm rabbits received intratracheally saline (control) or S-LPS and were ventilated with 100% oxygen. After 30 min, LPS-treated animals received no treatment, or surfactant (200 mg/kg) without/with 3% PxB; controls received the same dose of surfactant. Animals were ventilated for further 2 h. In vitro, addition of 5% S-LPS to surfactant increased minimum surface tension (γmin) and addition of 1–3% PxB to surfactant/S-LPS mixture restored γmin to low values. Animals only given S-LPS had lower lung compliance and lung gas volume (LGV) compared to surfactant groups. Treatment with surfactant/PxB, but not with surfactant only, restored LGV. Addition of PxB to the surfactant increased the alveolar expansion. S-LPS interferes with surface activity of the pulmonary surfactant and PxB improves the resistance of surfactant to LPS-induced inactivation. In our neonatal model of respiratory distress syndrome surfactant gives positive response even in simultaneous exposure to S-LPS, when enriched with PxB.

## Introduction

The interface between respiratory system and external environment is permanently exposed to the air- or bloodborne toxins. Lipopolysaccharide (LPS), also called endotoxin, is the major outer surface membrane component present in almost all Gram-negative bacteria^1^. LPS binds to toll-like receptors (TLRs) on cellular membranes and initiates the cascades of pro-inflammatory and pro-oxidative pathways^2^. In the lungs LPS induces damage to pulmonary tissue with influx and activation of neutrophils^3^, vascular-to-alveolar protein leakage and edema possibly leading to indirect surfactant inactivation, and cellular apoptosis and necrosis followed by respiratory failure. In addition to the effects mediated via TLRs LPS also directly inactivates pulmonary surfactant, a lipoprotein material coating the inner surface of the alveoli and small airways^4^.

Prematurely born babies often develop respiratory distress syndrome (RDS) due to reduced amount of endogenous surfactant^5^. In that situation the presence of LPS in respiratory system can worsen the adaptation after birth. Inflammatory response of the immature lung to injury or infection may exacerbate the severity of chronic lung disease and thus an early and efficient treatment is crucial for proper postnatal lung growth and development^6^. The management of neonatal RDS is targeted to maximize survival whilst minimizing potential adverse effects including bronchopulmonary dysplasia. The main causative therapy of RDS is surfactant replacement^7^. Exogenous surfactant administration has been of therapeutic benefit also in experimental LPS-induced lung injury^8–10^, although possible surfactant inactivation by LPS should be considered.

Surfactant inhibition in the alveoli can be overcome by treatment with animal-derived surfactant preparations or with synthetic surfactants based on SP-B and SP-C analogues and probably more resistant to inactivation^11,12^. Another strategy is to use additives to existing surfactant preparations such as dextran^13^ or polymyxin B (PxB)^14^. PxB is the antimicrobial peptide used in clinical practice to treat infections by resistant Gram-negative bacteria. It binds *Escherichia coli* lipopolysaccharides and prevents inflammatory activation^15^. It also mimics some activities of a specific surfactant protein B^16^, stabilizes surfactant multilayers and is able to increase the resistance of exogenous surfactant to serum albumin^17^, meconium^14^ and LPS in vitro^18^.

On the basis of the observations summarized above, administration of surfactant mixed with PxB would seem a rational approach to the management of lung injury caused by lung immaturity and LPS administration. Surfactant can improve spreading of additives and help to deliver them directly to the lung, thereby increasing local concentrations and reducing systemic side effects^19^. Surfactant/PxB mixture has been effective in animals with acute respiratory distress syndrome due to albumin leak^17^ and prevented bacterial growth in neonatal *E. coli* pneumonia of rabbits^20^, but has not been evaluated in other models of neonatal lung disease. The aims of the present study were to investigate, in captive bubble surfactometer, the interplay between surfactant, smooth LPS and PxB and to evaluate in a newly established double-hit neonatal rabbit model connecting lung immaturity and inflammatory response, the effect of PxB for restoring surfactant activity.

## Material and methods

### In vitro study

#### Surfactant

Modified porcine surfactant poractant alfa (Curosurf, Chiesi Farmaceutici, Parma, Italy) was used. For in vitro experiments it was diluted in saline (0.9% NaCl) to phospholipid (PL) concentrations of 3 and 5 mg/ml.

#### Lipopolysaccharide (LPS)

Purified lyophilized phenol extract of *Escherichia coli* (O55:B5, Sigma, St. Louis, Mo., USA), containing lipid A moiety linked to an antigenic *O*-polysaccharide indicating smooth S-LPS, was dissolved in saline to a concentration of 1 mg/ml and frozen in aliquots. For in vitro experiments it was diluted in saline (0.9% NaCl) and added to surfactant at concentration of 1, 3 and 5%.

#### Polymyxin B (PxB)

PxB Sulfate was purchased from Sigma (St. Louis, Mo., USA) and added to surfactant or surfactant/S-LPS mixture in concentrations of 0.5, 1, 2 and 3% (by mass of the surfactant PL).

#### Exposure of surfactant to LPS

Inactivation of surfactant was tested by addition of S-LPS to surfactant at different concentrations. Samples of surfactant with/without PxB were incubated at 37 °C for 30 min prior to measurement of dynamic surface tension.

#### Evaluation of surface activity by a captive bubble system

Surface activity of surfactant samples was measured by a captive bubble system (CBS)^21^. The test chamber was initially filled with 10% sucrose in saline. Two microliters of surfactant were injected into the sample chamber and allowed to migrate by buoyance to the agarose ceiling. An air bubble was then placed under the ceiling in contact with the surfactant preparation, and surface tension was measured from the time of bubble insertion. After 5 min of adsorption, the sample chamber was sealed, and the quasi-static cycling was initiated. The bubble was compressed stepwise until the surface tension ≤ 5 mN/m was reached and thereafter expanded to the initial size. Surface tension was measured for cycle 1, 3 and 5 and each quasi-static cycle was 2 min. Minimum and maximum surface tension were estimated as well as compression needed to reach γmin of 5 mN/m (area%). Mean values were obtained from 3 to 5 observations for each sample.

### In vivo study

#### General design of animal experiments

The experiments were performed on 69 preterm newborn rabbits (New Zealand White) from ten litters obtained by Caesarean section at a gestational age of 27 days (term, 31 days). As described elsewhere [e.g. Ref.^17^], after delivery, the animals were anesthetized with an intraperitoneal injection of pentobarbital sodium (APL, Stockholm, Sweden; 6 mg/ml; dose 0.1 ml), tracheotomized, paralyzed with intraperitoneal mixture of ketamine hydrochloride (Ketaminol, Intervet AB, Stockholm Sweden; 20 mg/kg) and medetomidine hydrochloride (Domitor, Orion Pharma, Espoo, Finland) (0.1 mg/kg) and kept in plethysmograph boxes at 37 °C. They were mechanically ventilated in parallel with a modified Servo-Ventilator (900B, Siemens-Elema, Solna, Sweden) with 100% oxygen, frequency of 40 breaths/min, an inspiration-to-expiration ratio of 1:1 and positive end-expiratory pressure (PEEP) 3 cm H_2_O. To open up the lungs, peak inspiratory pressure (PIP) was first set to 35 cm H_2_O for 5 inspirations. Then the pressure was lowered to obtain tidal volumes of 6–8 ml/kg of body weight (b.w) by individual adjustment of PIP. In animals whose lungs did not open a maximum pressure (PIP) of 25 cm H_2_O was used even if desired tidal volume was not reached.

**Figure 1:**
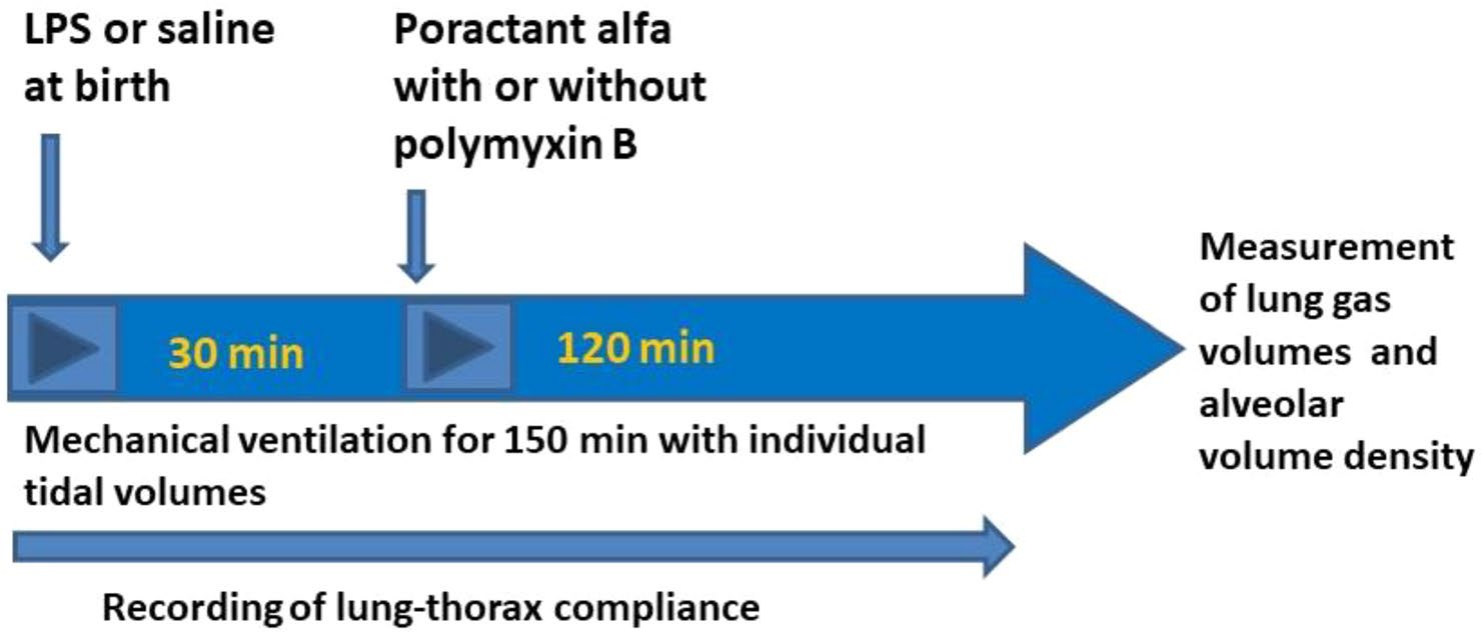
Schematic diagram of double-hit model of lung immaturity and acute lung injury by bacterial lipopolysaccharide (LPS) instillation in premature newborn rabbits and the overview of experimental protocol.

The animals were randomized to receive at birth (Fig. 1) via tracheal tube, S-LPS at a concentration of 0.25 mg/ml, volume dose 2 ml/kg to induce lung injury, or saline at the same volume dose and the animals were ventilated for 30 min (see above). After that the pups received intratracheally poractant alfa (80 mg/ml, 2.5. ml/kg) or the same dose of poractant alfa with 3% PxB and were further ventilated for 2 h. Animals receiving only LPS at birth served as negative controls and those receiving saline and poractant alfa as positive controls. The respiratory parameters were registered at 15, 30, 45, 60, 90, 120 and 150 min of mechanical ventilation.

At the end of the experiment the tracheal tube was clamped at end-expiration and the trachea ligated. Animals were overdosed by intraperitoneal injection of pentobarbital (60 mg/ml, 0.1 ml). The abdomen was opened and the diaphragm inspected for evidence of pneumothorax. The lungs were excised and weighed.

#### Lung function measurement

Peak inspiratory pressure was recorded with a pressure transducer (EMT34). Tidal volumes were recorded with a Fleisch-tube and recording system PowerLab 4/20 (ADInstruments, Oxfordshire, UK). Lung-thorax compliance was derived from recordings of tidal volume and the airway pressure gradient (PIP–PEEP) and expressed as ml/kg cm H_2_O^22^.

#### Lung-gas volume measurement

Lung-gas volume expressed in ml/kg of body weight was determined by water displacement technique described elsewhere^20,22^.

#### Morphometric examination of lungs

The whole lungs were fixed by immersion in 4% formalin and embedded in paraffin. Transverse sections from all lobes were stained with hematoxylin and eosin. Volume density of alveolar spaces was measured by computerized image analysis^23^ using total parenchyma as reference volume. The morphometric examination was blinded, i.e., the examiner was unaware of the experimental conditions of individual animals.

#### Ethical approval

The animal experiments have been performed in accordance with EU Directive 2010/63/EU for animal research and was approved by Local Ethical Committee for Animal Research, Stockholms Norra Djurforsöksetiska Nämnd No. N174/14.

#### Statistics

Data are shown as mean ± SD, or as median [range]. The statistical program STATISTICA version 9.1 (StatSoft, Tulsa, OK) was used for data analysis. The differences between groups were analyzed by one-way ANOVA followed by Neuman-Keuls’ post hoc test. Fisher's exact test was used to examine an association between pneumothorax and the treatment group. A *P* value ≤ 0.05 was regarded as statistically significant.

## Results

### In vitro study

#### Surface properties of poractant alfa in the presence of LPS

**Figure 2:**
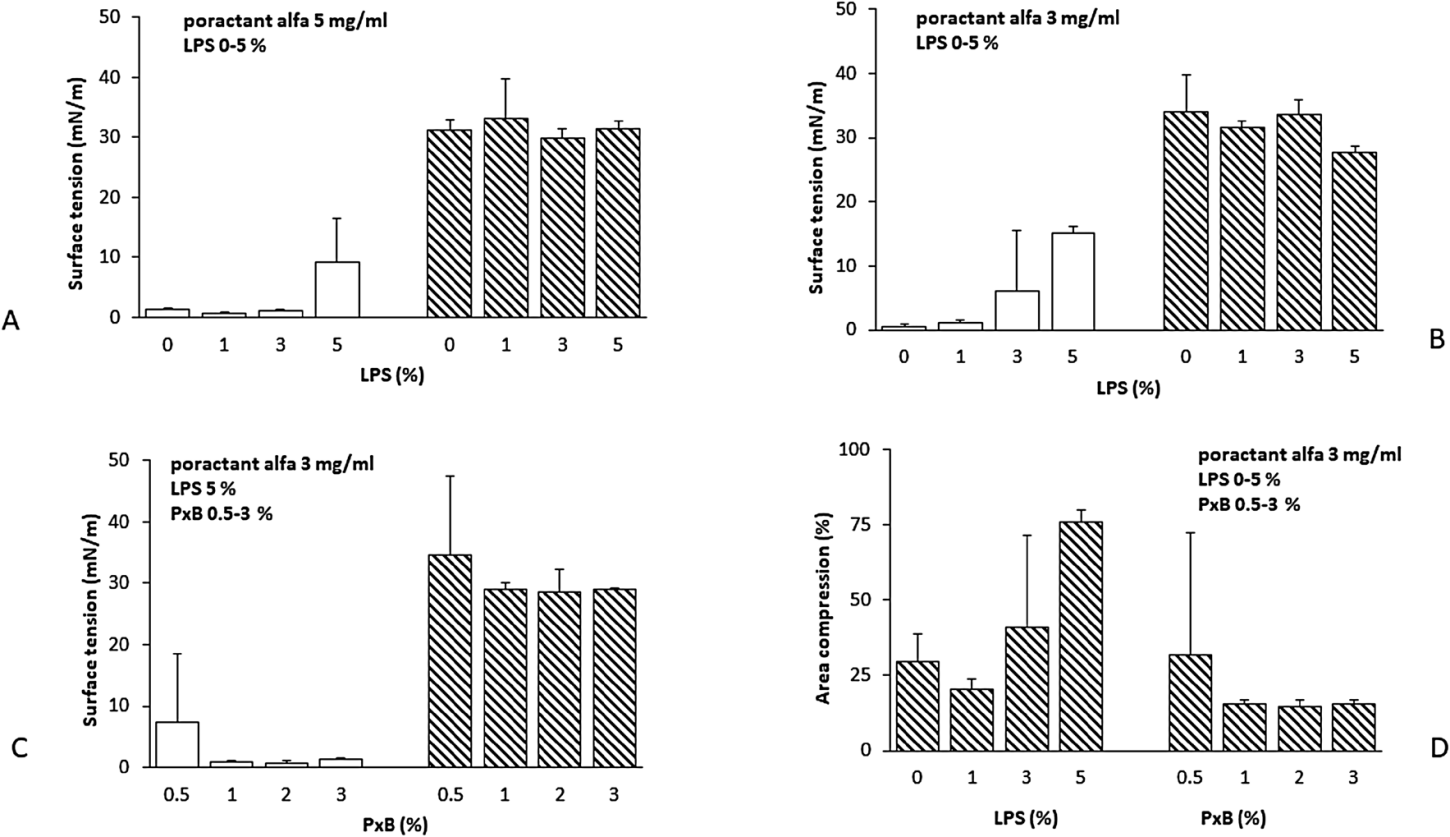
(**A**–**D**) Dynamic surface properties of poractant alfa at 5 mg of PL/ml without and with LPS at 1, 3 and 5% (**A**); poractant alfa at 3 mg of PL/ml without and with LPS at 1, 3 and 5% (**B**); poractant alfa at 3 mg of PL/ml with LPS at 5% and PxB at 0.5–3% (**C**) at minimum (empty columns) and maximum (striped columns) bubble size. Area of compression needed to reach minimum surface tension of 5 mN/m of poractant at 3 mg PL/ml and with LPS at 1, 3 and 5% (2D left), and poractant alfa/LPS 5% with PxB at 0.5, 1, 2 and 3% (2D right). All values were obtained during the 5th cycle in the captive bubble surfactometer and are shown as mean ± SD.

**Figure 3:**
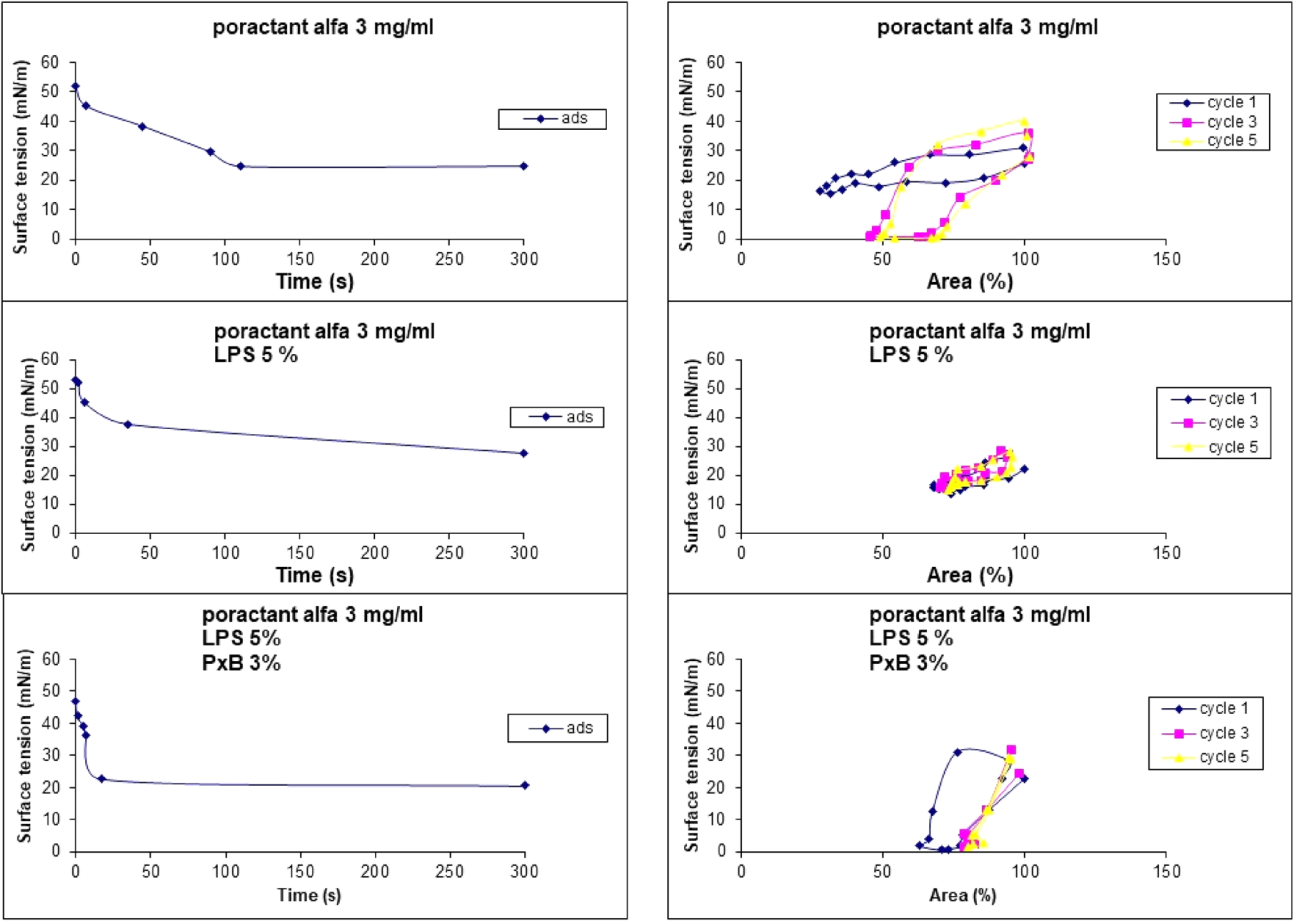
Representative diagrams of the behavior of the poractant alfa at 3 mg/ml (up), poractant alfa 3 mg/ml and 5% LPS (middle) and poractant alfa 3 mg/ml, 5% LPS and 3% PxB (down) in captive bubble surfactometer. Speed of absorption at the surface of the air bubble (left) and surface tension at 1st, 2nd and 5th cycle during compression (right).

Different concentrations of poractant alfa were tested in order to find the lowest concentration of PL at which the surfactant had a minimum surface tension (ƴmin) ≤ 5 mN/m during the 5th cycle in the captive bubble surfactometer and was sensitive to inactivation by LPS. Using a surfactant concentration of 5 mg/ml this situation occurred at an LPS concentration of 5% (related to surfactant PL) (Fig. 2A). This finding was not consistent as indicated by large sample variability. At reduced PL concentration to 3 mg/ml poractant alfa reached ƴmin of 0.6 ± 0.1 mN/m which gradually increased with increasing LPS concentration (Figs. 2B, 3). In the presence of 5% LPS ƴmin was 15.2 ± 0.5 mN/m indicating low surface activity. LPS had no effect on maximum surface tension (ƴmax) (Figs. 2A, B, 3).

#### Effect of PxB on poractant alfa inactivated by LPS

A surfactant concentration of 3 mg/ml and LPS 5% (w/w of surfactant PL) was chosen to study the effect of PxB on surfactant inhibition by LPS. Dynamic surface properties of poractant alfa were not improved by PxB 0.5%. Increasing PxB to 1–3% improved the ability of poractant alfa to reach low surface tension in presence of LPS (Fig. 2C).

The results on surface tension are corroborated by calculation of area of compression. It is measured as the difference between bubble area at maximum surface tension and when surface tension is less or equal to 5 mN/m. A surface tension of 5 mN/m could only be obtained for poractant alfa 3 mg/ml with 5% LPS using an area compression of 76 ± 7.7%. Addition of PxB at 1–3% to the poractant alfa/LPS mixture improved the in vitro surface activity (Fig. 2D).

Taken together, surfactant at low phospholipid concentration is more susceptible to LPS inactivation. At 3 mg of PL/ml, poractant alfa is inactivated by LPS 3% and higher. Already addition of 1% PxB to the poractant alfa/LPS mixture makes it more resistant to inactivation.

### In vivo experiments

The concept of animal experiments was based on the results of in vitro study. The animals received at birth saline or LPS at a dose 500 μg/kg b.w. and were after 30 min treated with poractant alfa 200 mg/kg without or with 3% PxB (w/w) or had no further treatment. Of 69 premature rabbits used in the experiments, data of 57 were statistically processed. Twelve animals had to be excluded from final data analysis based on pneumothorax within the period of observation. No differences in body weight and incidence of pneumothorax were present between the groups (Table 1).

**Table 1:** Body weight, numbers, incidence of pneumothorax, lung gas volume and alveolar volume density in preterm newborn rabbits exposed to saline or LPS, and treated with exogenous surfactant with or without PxB.

Treatment	Body weight (g)	n	Incidence of pneumothorax	Lung gas volume (ml/kg)	Alveolar volume density (%)
Saline/poractant alfa	27.2 ± 3.9	14	2	4.6 ± 1.5	59.1 ± 4.0
LPS	29.5 ± 4.2	15	2	0.4 ± 0.2	27.7 ± 3.3
LPS/poractant alfa	29.1 ± 4.7	13	2	2.7 ± 1.0	45.2 ± 3.4
LPS/poractant alfa/PxB	28.6 ± 5.1	15	6	3.9 ± 1.3	51.4 ± 3.8

Values of body weight, lung gas volume and alveolar volume density are mean ± SD; *n*, number of animals included in final statistical analysis.

Positive controls received saline at birth and were treated with poractant alfa. LPS exposed and non-treated animals were used as negative controls. Statistical analysis by one-way ANOVA for lung gas volume: LPS vs. saline/poractant alfa and LPS/poractant alfa/PxB, P < 0.001 and LPS vs. LPS/poractant alfa, P < 0.01; LPS/poractant alfa vs. saline/poractant alfa P < 0.01; alveolar volume density for all between-group differences, P < 0.001.

#### Lung-thorax compliance

**Figure 4:**
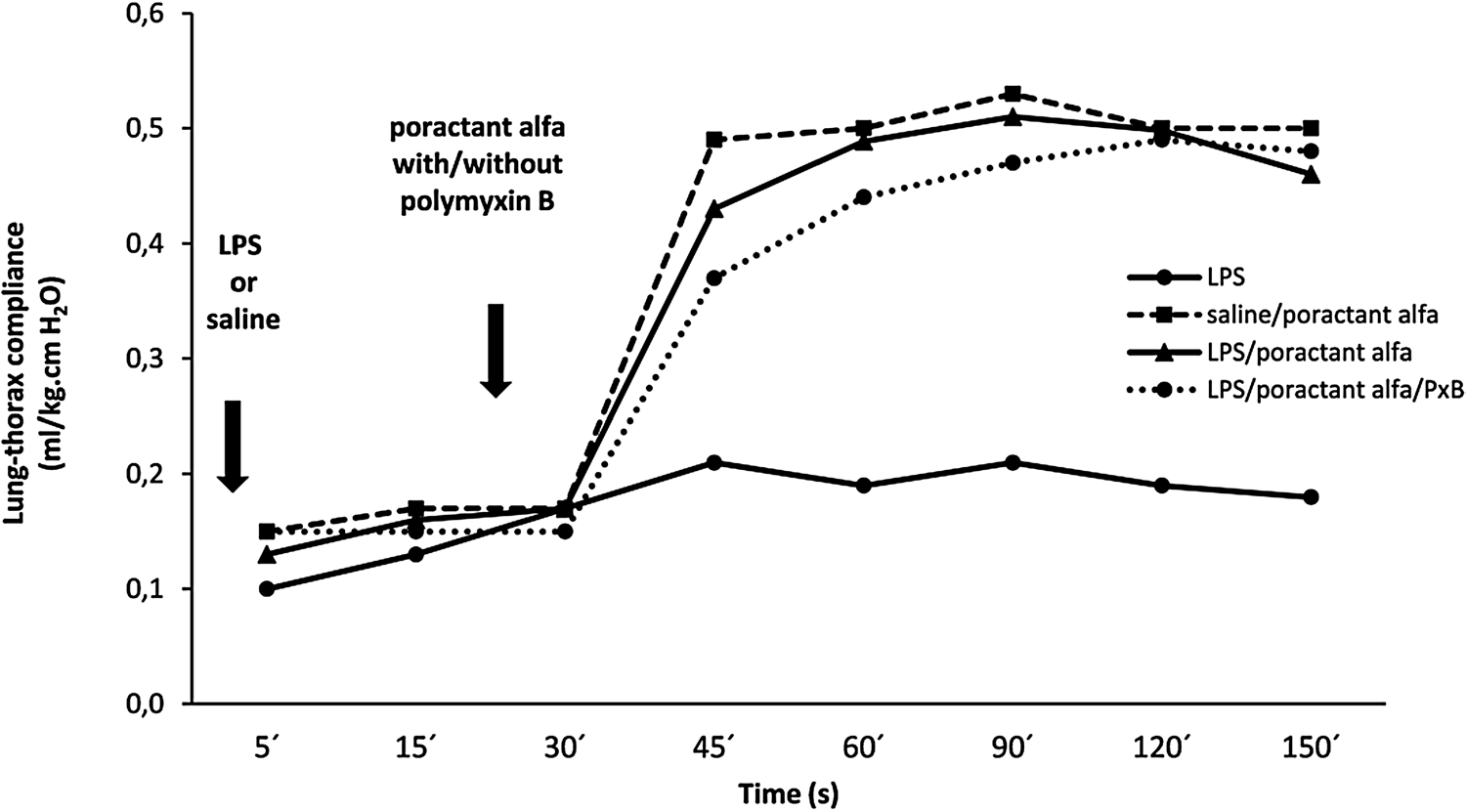
Lung-thorax compliance during the experiment in preterm newborn rabbits exposed to LPS (negative control) or saline (positive control) at delivery and treated with poractant alfa with or without PxB at 30 min of ventilation. Statistical analysis: at all time points since 45th min, all groups > LPS; P < 0.001; at 45th min, LPS/poractant alfa/PxB < saline/poracant alfa, P < 0.05.

After LPS or saline lung-thorax compliance (LTC) was low in all groups (~ 0.10–0.15 ml/kg.cm H_2_O) with no differences until treatment with surfactant with or without PxB. Fifteen minutes after this treatment, i.e. 45 min after beginning of ventilation and further on, LTC was higher in all surfactant-treated groups in comparison with non-treated animals with LPS (all P < 0.001) (Fig. 4). At 45th min animals receiving poractant alfa/PxB mixture had lower compliance than positive control (saline/poractant alfa; *P* < 0.05) (Fig. 4).

#### Lung gas volume and alveolar volume density

Lung gas volume (LGV) at the end of experiment was significantly higher in all treatment groups in comparison with negative control receiving only LPS (P < 0.001). Mean lung gas volumes in LPS/poractant alfa group without PxB were significantly lower than in saline/poractant alfa group (P < 0.05) while there was no significant difference between positive control (saline/poractant alfa) and LPS/poractant alfa/PxB group. The animals receiving LPS and treated with poractant alfa and PxB tended to have higher LGV than those without PxB (P > 0.05) (Table 1).

**Figure 5:**
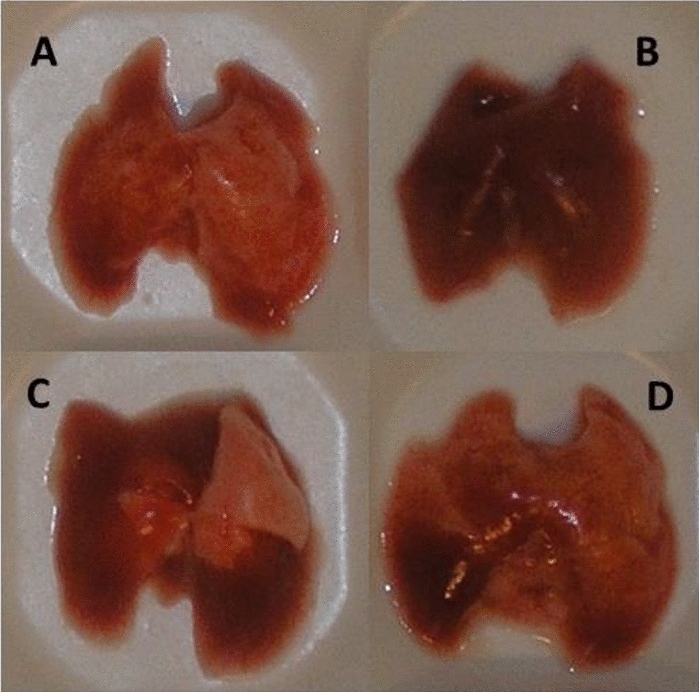
Representative macroscopic appearance of the lungs in preterm newborn rabbits after administration of saline and poractant alfa (**A**), LPS and no further treatment (**B**), LPS and poractant alfa (**C**), and LPS and poractant alfa with PxB (**D**).

Values on LGV correspond to macroscopic picture of the lungs (Fig. 5) and are confirmed with data on alveolar volume density which indicate the grade of alveolar expansion (Table 1).

Premature animals with LPS (negative controls) had significantly lower values of alveolar volume density than surfactant-treated groups (all P < 0.001). Alveolar volume density was significantly higher in group treated with LPS/poractant alfa/PxB mixture than with LPS/poractant alfa only (P < 0.001) (Table 1).

## Discussion

In this study we verified the hypothesis that PxB by protecting the exogenous surfactant may facilitate the response of immature lungs with LPS-induced lung injury to surfactant replacement therapy. The hypothesis was tested in a preterm rabbit model which combines respiratory distress syndrome of prematurity with LPS-induced lung damage.

First, we investigated using a captive bubble surfactometer the interaction of bacterial LPS with modified porcine surfactant (poractant alfa) and whether this interaction could change when PxB was added. In this study the consistent inhibition of surfactant at 3 and 5 mg of phospholipids/ml was achieved with 3% LPS (w/w to surfactant PL) (Fig. 2A,B). In the past, the interaction of LPS and the surfactant was studied preferentially in synthetic lipid monolayers [e.g. Refs.^24,25^]. In surfactant-like films, composed of either dipalmitoylphosphatidylcholine (DPPC) or DPPC/palmitoyloleoylphosphatidylglycerol/palmitic acid, LPS fluidized DPPC monolayers and prevented to achieve low surface tension. Our results are in accordance with recent experiments with pulsating bubble surfactometer when already 1% LPS (w/w) prevented surfactant at 1.75 mg PL/ml to reach low surface tension during area compression^18^. Polarizing microscopy and small-angle scattering after 2 h incubation of surfactant/LPS mixture at 37 °C revealed that LPS binds to the lipid bilayer. It disturbs lamellar packing in the dispersion of surfactant and causes a drop in the population of multilamellar structures^18^. Similar mechanism of surfactant inhibition can be expected in natural conditions in the respiratory system.

An animal model with single insult does not fully reflect the complex background of the diseases. Injury or infection may exacerbate the severity of chronic lung disease in predisposed babies, and lung inflammation and lung immaturity are the major risk factors for e.g., bronchopulmonary dysplasia^6,26^. This is why testing of treatment modalities of neonatal lung disorders requires the use of more complex models to reflect their multifactorial pathophysiology^27,28^. In our animal experiments we have mimicked lung injury in early postnatal period caused by multiple insult by intratracheal administration of LPS to preterm rabbits born in 27-day of gestation (term, 31 days). Immature newborn animals are a most relevant model of neonatal respiratory distress syndrome (RDS). They are lacking endogenous surfactant and fail to establish functional residual capacity at birth^29^. Instillation of LPS induces inflammation and further potentiates lung damage due to immaturity. The animal model used in the present study represents a new approach for evaluation of acute effects of surfactant substitutes with focus on their resistance to inactivation by LPS. Intratracheal administration of LPS leads to acute lung injury (ALI)/acute respiratory distress syndrome (ARDS). It causes neutrophilic inflammatory response with increase in intrapulmonary cytokines and because of high reproducibility it is used widely, especially in rodents^30^. The dose of LPS used in the present study was 500 μg/kg of b.w. Data regarding the type (smooth vs. rough) of LPS and its appropriate dosage differ. In numerous experimental studies the LPS was intratracheally administered in a wide range of 5–5000 µg/kg of b.w.^31–33^. The dose chosen here was based on our pilot experiments in adult rats that have developed lung edema, local and systematic inflammation and oxidative stress after LPS 500 μg/kg of b.w.^34^.

In the alveoli LPS combines with endogenous surfactant and/or with exogenous surfactant preparations instilled into the lungs. Among others, this process is facilitated by the ability of SP-C to bind to the lipid A part through N-terminal domain^35^. In the late 80’s it was shown for the first time that LPS creates complexes with surfactant and alters its properties contributing to changes seen in some kinds of pneumonia^36,37^. As mentioned above, LPS disturbs lamellar structure of surfactant by swelling and thus affects its biophysical and physiological properties. Moreover, in the lungs LPS binds to the toll-like receptor (TLR) complex CD14/TLR4/MD-2 on cellular membranes. Increase in transcription factor NF-κB, and activation of both pro-inflammatory and pro-oxidative pathways contribute to tissue damage and increase permeability of alveolar-capillary barrier which further potentiates surfactant inactivation and lung injury^38^.

Endotoxin-induced lung injury can be treated by intratracheal administration of exogenous surfactant. The beneficial effect of such treatment has been previously confirmed for both natural porcine and synthetic surfactants. The positive effect of the natural surfactant was more pronounced than that of the synthetic one^10^. Earlier the synthetic preparation based on the peptide KL_4_ was used in a similar model in mice^9^. In those studies, the effect of surfactant treatment was not verified until 24 and 72 h, respectively, after LPS administration. There is only one study in which the modified exogenous surfactant poractant alfa was given to spontaneously breathing rats with LPS-induced early-stage ARDS^8^. Poractant alfa was administered by an intratracheal bolus of 62.5, 125 or 250 mg/kg following an intratracheal lipopolysaccharide (1.6 mg/kg). These treatments improved respiratory frequency and decreased mortality, pulmonary edema and inflammation.

In the present animal experiments poractant alfa was administered at the recommended clinical dose (200 mg/kg b.w.) alone or in combination with PxB. Surfactant treatment (poractant alfa only) in animals with LPS restored lung-thorax compliance by the same degree as in animals receiving saline (positive control). Lung gas volume (LGV) and alveolar volume density were significantly higher than in animals serving as negative control reflecting the fact that surfactant was inactivated by LPS and thus not able to stabilize the lungs at the end of expiration. It corresponds to the situation in vitro experiments documented in Fig. 2A,B.

Addition of PxB to poractant alfa made the surfactant more resistant to inactivation to LPS as demonstrated by restoration of low surface tension in the captive bubble surfactometer (Fig. 2C). It occurred at 1% PxB*.* This effect was seen earlier in another in vitro model^18^ or in the model of leaking plasma proteins where in vitro addition of 2% PxB improved surface activity of poractant alfa at low concentration and increased its resistance to inactivation by albumin^17^.

Surfactant/PxB mixture was tested previously in an animal model of alveolar overflooding by plasma proteins^17^ and in neonatal *Escherichia coli* pneumonia where it had antimicrobial effect and prevented systemic spreading of *E. coli*^20^. In alveolar type II culture this mixture had no negative effect on cell viability and surfactant exocytosis that encourage the intention to use surfactant as a vector for PxB administration^39^. In our neonatal double-hit model of lung immaturity and LPS-induced acute lung injury treatment with poractant alfa/PxB, but not with poractant only, restored lung gas volumes and alveolar volume density to the degree comparable with positive control animals (saline/poractant alfa) (Table 1, Fig. 5).

Treatment with poractant alfa/PxB did not further improve lung-thorax compliance, but improved expansion as documented by LGV and alveolar expansion pattern evaluated at the end of experiments. This finding confirms that LGV better reflects the quality of surfactant material and its ability to stabilize the lungs at end-expiration.

On the other hand, preterm animals with LPS treated with poractant alfa/PxB mixture tended to have higher incidence of pneumothorax in comparison with the other groups in period of observation (Table 1). A higher incidence of pneumothorax, although not statistically significant, may be indicative of excessive lung expansion. This finding is in contrary to our previous study where the incidence of pneumothorax was reduced in premature newborn rabbits treated with poractant alfa at 80 mg/kg + PxB undergoing prolonged ventilation^17^. The animals in both studies were delivered at 27th day of gestation and have been very preterm. The mechanism of superposed lung damage was different. In the previous study we mimicked the situation of impaired alveolar-capillary membrane and flooding of the alveoli by albumin. Albumin interacts with pulmonary surfactant and itself has no adverse effect on lung tissue, thus the damage was relatively mild. In the present study besides interacting with surfactant LPS also initiated inflammatory response leading to intense lung injury. The lung tissue was probably more vulnerable which may indicate that the alveoli are more sensitive for treatment with the active surfactant/PxB mixture giving a high alveolar expansion. The positive effect of PxB added to exogenous surfactant in vivo is in accordance with the results obtained by captive bubble surfactometry. LPS interferes with poractant alfa and leads to its inactivation which is prevented by addition of PxB. In vitro effect of PxB is attributed to its ability to cross-link phospholipid membranes and to inhibit structural disarrangement induced by LPS. Cationic molecule of PxB acts as an inhibitor of LPS-induced structural changes of surfactant. Due to charges compensation it restores original lamellar packing of surfactant^18^.

The interaction between PxB and LPS should not be underestimated either. It was intensely studied because of its importance in treatment of Gram-negative infections^40^. PxB induces LPS aggregation in a concentration-dependent manner. According to Domingues et al.^41^ PxB mechanism of action at the molecular level involves a first step of electrostatic approach toward LPS. In the lungs of preterm animals with LPS administration it is probably that PxB binds to *E. coli* LPS^15^ and thereby mitigates inflammatory response and prevents LPS/surfactant interaction.

## Conclusion

In summary, LPS interferes with surface-active properties of the pulmonary surfactant and prevents the alveoli from reaching low surface tension during expiration. A cationic antibiotic PxB improves the resistance of surfactant to inactivation caused by LPS. Administration of LPS to premature newborn rabbits represents a model of two-hit lung injury which may better reflect clinical situation resulting from lung immaturity and inflammatory response. Therapy with exogenous surfactant improves lung function, but is not enough to fully resume lung gas volume and alveolar volume density. Enrichment of exogenous surfactant with PxB potentiates the effect of surfactant therapy and gives positive response even in simultaneous exposure to LPS. The results indicate the synergic effect of surfactant and PxB in this neonatal model of RDS and a possible potential of this combination therapy in premature infants with LPS-induced lung injury.
